# The Stiffness and Damping Characteristics of a Rubber-Based SMA Composite Shock Absorber with a Hyper-Elastic SMA-Constitutive Model Considering the Loading Rate

**DOI:** 10.3390/ma17164076

**Published:** 2024-08-16

**Authors:** Yizhe Huang, Huizhen Zhang, Qiyuan Fan, Qibai Huang, Lefei Shao, Xin Zhan, Jun Wang

**Affiliations:** 1 Hubei Key Laboratory of Modern Manufacturing Quality Engineering, School of Mechanical Engineering, Hubei University of Technology, Wuhan 430068, China; yizhehuang@hbut.edu.cn (Y.H.); 102210107@hbut.edu.cn (H.Z.); 102310147@hbut.edu.cn (Q.F.); 2 State Key Laboratory of Digital Manufacturing Equipment and Technology, Huazhong University of Science and Technology, Wuhan 430074, China; qbhuang@hust.edu.cn (Q.H.); m202170399@hust.edu.cn (L.S.); d202287005@hust.edu.cn (X.Z.); 3 Dongfeng Liuzhou Motor Co., Ltd., Liuzhou 545005, China

**Keywords:** shock absorber, shape–memory alloy, improvement of Auricchio constitutive model, stiffness–damping characteristics

## Abstract

Shock absorbers are essential in enhancing vehicle ride comfort by mitigating vibrations. However, traditional rubber shock absorbers are constrained by their fixed stiffness and damping properties, limiting their adaptability to varying loads and thus affecting the ride comfort, especially under extreme road conditions. Shape Memory Alloys (SMAs), known for their intelligent material properties, offer a unique solution by adjusting stiffness and damping in response to temperature changes or strain rates, making them ideal for advanced vibration control applications. This study builds upon the Auricchio constitutive model to propose an enhanced SMA hyper-elastic constitutive model that accounts for different loading rates. This new model elucidates the impact of loading rates on the stiffness and damping characteristics of SMAs. Additionally, we introduce an innovative circular rubber-based SMA composite vibration reduction structure. Through a parameterized model and finite element simulation, we comprehensively analyze the stiffness and damping properties of the composite damper under various loading rates and harmonic excitations. Our findings suggest a novel approach to improving the vehicle ride comfort, offering significant potential for engineering applications and practical value.

## 1. Introduction

The suspension system of a commercial vehicle cab reduces vibration transmission from the frame to the cab by adjusting the stiffness and damping, thereby improving ride comfort. As an important component of the suspension system, shock absorbers can effectively attenuate the impact of road surface and load on the frame and body of the car while driving, improving the smoothness and comfort of the car. In recent years, more and more scholars and enterprises have begun to pay attention to and increase investment in the research and development of shock absorbers to improve their damping performance [1,2]. Smith [3] first proposed a commercial vehicle cab suspension system. Afterward, Schneider [4] introduced hydraulic devices into the shock absorber structure and proposed an active control scheme to reduce the cab vibration. Brown and Wilson [5] studied the frequency-response characteristics and vibration transmission rate of cab dampers under different road conditions through simulation and experimental methods, laying the foundation for the practical application of this technology. Yamamoto et al. [6] proposed a design scheme for actively controlling electromagnetic dampers, improving driver comfort and work efficiency. Sindgikar et al. [7] investigated the effect of commercial vehicle cab pitch angle on the ride comfort and aimed to control the pitch angle by optimizing the stiffness and damping parameters of the shock absorber to improve ride comfort effectively. Wang [8] addressed the issue of the poor performance of traditional ceiling algorithms in semi-active damper control by adopting a linear quadratic optimal algorithm to improve the control strategy. Boreiry et al. [9] improved the Bouc–Wen model of the magneto-rheological damper in the cab, effectively enhancing the accuracy of numerical calculations for the magneto-rheological damper. Sim et al. [10] established a 14-degree-of-freedom vibration model for the cab damper, significantly improving the accuracy of the coupling calculation between chassis suspension and cab suspension.

However, with the increasing demand for ride comfort, traditional suspension systems cannot adequately meet vibration reduction requirements. Rubber materials are widely used in vibration reduction and isolation design in fields such as automobiles, aerospace, and construction due to their excellent vibration reduction and energy dissipation characteristics. Compared to the initial rigid fixation of the cab to the frame, introducing rubber shock absorbers has greatly improved ride comfort.

Li and Wei [11] conducted tensile and hardness tests on rubber materials and studied the rubber characteristics suitable for heavy-duty commercial vehicle shock absorbers. Chen and Zhang [12] consider multiple factors, such as vibration reduction effect, durability, and cost, and use optimization methods, such as genetic algorithms, to seek the optimal combination of structural parameters for shock absorbers. They also optimize the design of rubber shock absorbers for heavy-duty commercial vehicles, thereby improving the vibration reduction efficiency of commercial vehicle shock absorbers. In addition, researchers also focused on the dynamic response characteristics of rubber dampers under different road conditions to analyze their impact on the vehicle’s driving stability and ride comfort. Wang and Liu [13] investigated the effects of stiffness, geometric shape, and structural parameters of rubber materials on the smoothness and handling of commercial vehicles through a finite element analysis and experimental testing. They also researched the dynamic response characteristics of commercial vehicle rubber dampers, providing an important theoretical basis for improving commercial vehicle dampers. Zhang and Wang [14] proposed a method for predicting and evaluating the fatigue life of rubber shock absorbers by combining experiments and numerical simulations. Analyzing the fatigue fracture characteristics and stress distribution of materials can effectively predict the fatigue damage that may occur during the long-term use of shock absorbers, providing an important reference for the durability design of rubber shock absorbers. Gao and Zhou [15] analyzed the performance of rubber dampers under actual working conditions through long-term road testing and data collection, providing experimental references for their application in commercial vehicles. In summary, current research on rubber shock absorbers primarily focuses on identifying rubber materials with enhanced performance and durability, as well as conducting in-depth studies on the dynamic characteristics of the rubber shock absorbers to optimize their parameters and improve vibration reduction outcomes. However, due to the inherent structure and molecular chain properties of rubber materials, their stiffness and damping are relatively fixed, leading to suboptimal vibration reduction [16]. Therefore, it is particularly important to study how to use intelligent materials to design the composite dampers with a wide range of adjustable stiffness and damping and good vibration reduction effects.

Shape Memory Alloys (SMAs) are intelligent materials capable of restoring their original shape in response to temperature or stress changes. They possess two key characteristics: the shape–memory effect and super-elasticity [17,18]. Due to these unique properties, the SMAs are frequently used in the design of intelligent shock absorbers. This application has garnered significant attention in both domestic and international research, and the use of SMA shock absorbers continues to expand [19,20].

Chowdhury and Sehitoglu [21] studied the applicability of the SMA atomic model at the lattice scale and verified through numerical simulations that the functional fatigue of SMA is caused by the continuous slip accumulation at grain boundaries during the phase transition process. Mulay and Shmerling [22] used shape–memory alloy materials to design shock absorbers and investigated the effects of structural form, damping ratio, vibration frequency, and other parameters on vibration reduction. Raczka et al. [23] manufactured shock absorbers using shape–memory alloys and applied them to automotive suspension systems. By adjusting the working temperature of SMA shock absorbers, the real-time control of the stiffness of the automotive suspension system was achieved, thereby improving suspension performance and ride comfort. Childs and Greenwood [24] designed a semi-active control SMA suspension for heavy-duty commercial vehicles, demonstrating the effectiveness of SMA dampers in improving handling and stability through simulation analysis and experimental verification. Alaa [25] designed a new type of SMA damper and applied it to building structures to improve seismic performance during earthquakes. Zhou [26] installed the SMA dampers in bridge structures and evaluated their vibration suppression performance. The results showed that SMA dampers can effectively reduce bridge vibration, thereby enhancing structural stability and safety. Dutta and Majumder [27] developed a new type of SMA damper with damping control and applied it to mechanical processing equipment, significantly reducing vibration and improving processing quality and efficiency. Mani and Senthilkumar [28] created a dynamic SMA-based damper for pipeline vibration control, utilizing the characteristic of SMA in which its elastic modulus changes with temperature. By connecting the SMA springs in parallel using an electric current, the stiffness of the springs can be adjusted through the thermal activation, thus tuning the natural frequency of the dynamic damper and effectively reducing vibration energy within a specific frequency range.

This article takes into account the influence of the loading rate, based on the rubber and the improved Auricchio constitutive model, and supplemented by the ANSYS19.2 secondary development tool, to delve into the effect of different excitation amplitudes and frequencies on the stiffness and damping characteristics of rubber shock absorbers. A rubber-based SMA composite shock absorber structure was put forward; one potential application of the rubber-based SMA composite shock absorber proposed in this study is in the automotive industry. For example, it can be used in the suspension system of vehicles to improve the ride comfort and handling stability. The adjustable stiffness and damping characteristics of composite shock absorbers help to better adapt to different road conditions and driving scenarios, reducing the vibration and impact transmitted to the body and passengers. In addition, this type of shock absorber can also be applied to other fields that require vibration control and energy dissipation, such as aerospace. For example, in aerospace engineering, it can be used for landing gear or structural components to reduce vibration during the flight and landing. Its stiffness and damping characteristics under different loading rates and excitation amplitudes were analyzed in detail through the parametric modeling and finite element simulation, with the aim of enhancing the ride comfort of vehicles.

## 2. Improvement of SMA Hyper-Elastic Constitutive Model

The Auricchio model provides a mathematical description of the behavior of shape–memory alloys (SMAs) during the stress–strain transition process. This model utilizes the energy density function to depict the mechanical behavior of SMAs, integrating the elastic stress–strain relationship and phase transition dynamics. It effectively predicts the mechanical response of SMAs during both loading and unloading processes, encompassing hyper-elasticity and phase transitions. Within the range of SMA super-elastic loading and unloading, the stress–strain relationship can be expressed as follows:

(1) $$\sigma =D:\left(\varepsilon -{\dot{\varepsilon }}_{\mathrm{tr}}\right),$$

(2) $${\dot{\varepsilon }}_{\mathrm{tr}}=\dot{\xi }{\bar{\varepsilon }}_{u}\frac{\partial F}{\partial \sigma }.$$

In Formulas (1) and (2), σ and ε represent the stress and strain, respectively, generated within the loading and unloading range of SMA; D is the elastic stiffness tensor; ${\dot{\varepsilon }}_{tr}$ is the deformation strain tensor; $\dot{\xi }$ represents the hyper-elastic coefficient of SMA material; ${\bar{\varepsilon }}_{u}$ is the maximum stress during the loading process; and ∂F/∂σ represents the partial derivative of the load, with respect to stress, during the loading and unloading process.

This model provides only the most basic calculation framework for the super-elastic phase transition stage of SMAs and does not fully consider the influence of strain rate on the phase transition characteristics of SMAs. Consequently, the following will introduce the relationship between the stress increment and strain rate during the hyper-elastic phase transition stage, and improve the hyper-elastic Auricchio constitutive model of SMA.

**Figure 1:**
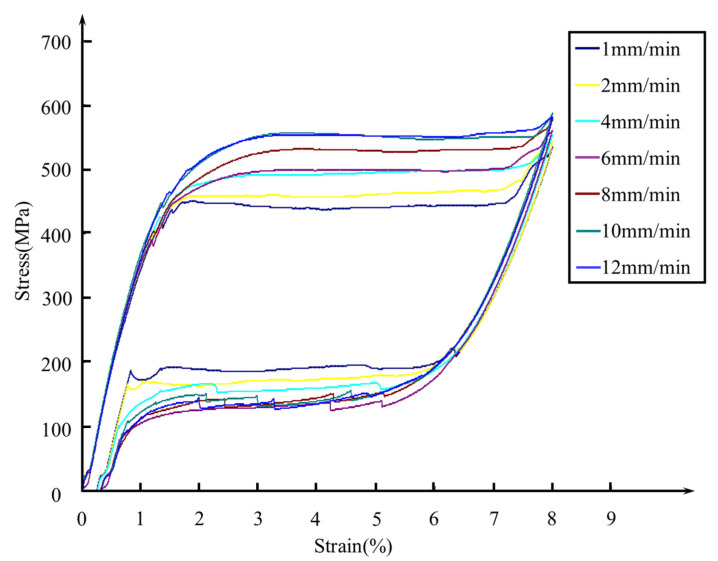
Stress–strain curves of SMA wire under different loading rates [29].

There is a notable difference in stress between the austenite and martensitic transformation stages during the loading and unloading processes of SMA at different loading rates. This difference is defined as stress increment and stress reduction. Displacement loading and unloading tests were conducted on a 100 mm-long Ni–Ti-shaped memory alloy wire specimen with a maximum strain amplitude of 8 % at loading rates of 1 mm/min, 2 mm/min, 4 mm/min, 6 mm/min, 8 mm/min, 10 mm/min, and 12 mm/min. Figure 1 displays the stress–strain curves of SMA wires at various loading rates.

**Figure 2:**
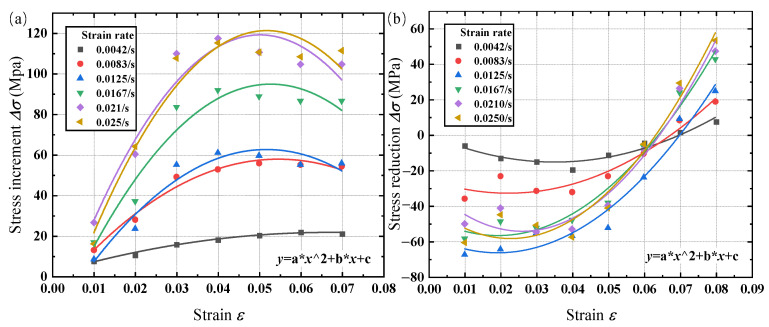
Fitting curves of stress changes and strain values under different strain rates at different stages: (**a**) loading; (**b**) unloading.

The stress increment and reduction data at different loading and unloading rates were processed using a benchmark rate of 1 mm/min or a strain rate of 2.1 × 10^−3^/s. Figure 2 displays the regression analysis of strain amplitude with the stress increment and reduction data.

Figure 2 shows the relationship between the stress increment, stress reduction, and strain at various strain rates after polynomial fitting. Equation (3) illustrates the fitting function:

(3) $$\Delta \sigma =a\varepsilon^{2}+b\varepsilon +c$$

Here, a, b, and c are the fitting parameters related to the strain rate.

**Figure 3:**
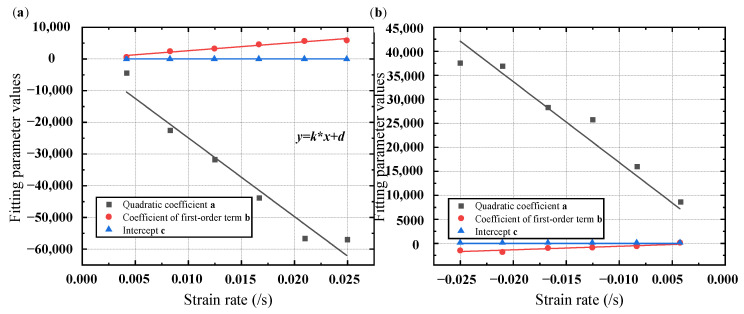
Linear fitting curves of different fitting parameters and strain rates at different stages: (**a**) loading; (**b**) unloading.

After noting the fitting parameters at various strain rates, linear fitting was performed with a zero intercept between the polynomial fitting coefficients and strain rates; Table 1, Figure 3, and Table 2 display the fitting results.

From Figure 3, it can be concluded that during the loading and unloading stages, as the strain rate increases, the quadratic coefficient a generally decreases, the first-order coefficient b generally increases, and the intercept c remains basically unchanged. This indicates that during the loading process, the greater the strain rate, the more significant the nonlinear relationship between stress increment and strain.

According to Table 2, during the loading and unloading stages, the quadratic coefficient a and the first-order coefficient b are positively correlated with the strain rate. That is, as the strain rate increases, the absolute values of the quadratic coefficient a and the first-order coefficient b will increase.

Through the polynomial fitting results of stress changes and strain values at different stages and strain rates, as well as the linear fitting results between the polynomial fitting parameters and strain rates, the relationship between the stress increment and strain at different stages and strain rates can be derived as follows:

(4) $$\Delta \sigma =\left\{\begin{array}{c} -2.49\times 10^{6}\cdot \dot{\varepsilon }\cdot \varepsilon^{2}+2.57\times 10^{5}\cdot \dot{\varepsilon }\cdot \varepsilon -1.34\times 10^{3}\cdot \dot{\varepsilon }\;Loading\\ -1.69\times 10^{6}\cdot \dot{\varepsilon }\cdot \varepsilon^{2}+7.38\times 10^{5}\cdot \dot{\varepsilon }\cdot \varepsilon -52.2\times 10^{3}\cdot \dot{\varepsilon }\;Unloading \end{array}\right.$$

Using Equation (4), the stress increment values correspond to each strain amplitude at different strain rates. By integrating those results with Equation (1), the stress during both the loading and unloading stages of the SMA can be updated. Additionally, Equation (5) offers the stress calculation formula for the improved Auricchio constitutive model.

(5) $$\sigma =D:\left(\varepsilon -{\dot{\varepsilon }}_{\mathrm{tr}}\right)+\Delta \sigma$$

## 3. Structural Design and Stiffness-Damping Characteristics Analysis of Rubber-Based SMA Composite Vibration Dampers

### 3.1. Analysis of Stiffness and Damping Characteristics of Rubber

**Figure 4:**
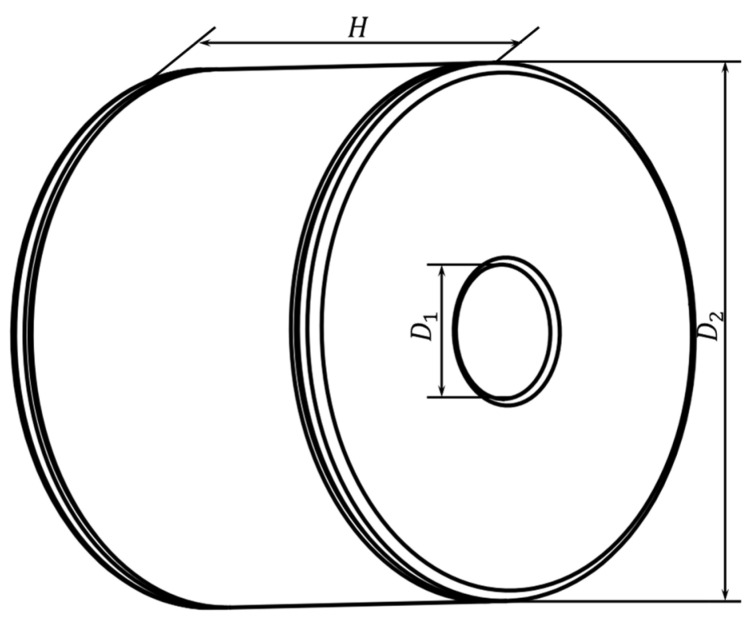
Structural diagram of the sleeve-type shock absorber.

The shock absorber used in this article is a traditional sleeve structure, as shown in Figure 4.

The rubber shock absorber has circular placement grooves that are 1 mm deep positioned on both sides. Table 3 lists the key structural characteristics. For it to work, the rubber shock absorber’s outside surface is attached to the frame support, and the driver’s cab fixing bolt is connected to it through the center through-hole. The driver’s cab-fixing bolt receives the excitation force from the engine and the road surface through the frame support, the rubber shock absorber, and dissipation.

The parameters of the hyper-elastic and viscoelastic constitutive models used in this article were determined based on the literature, as shown in Table 4 and Table 5.

Here, μ is the stiffness parameter of the material, α is the deformation tolerance index of the material, and D is the bulk modulus that characterizes the compressibility of the material.

Here, G is the spring elasticity coefficient, and τ is the damping coefficient.

**Figure 5:**
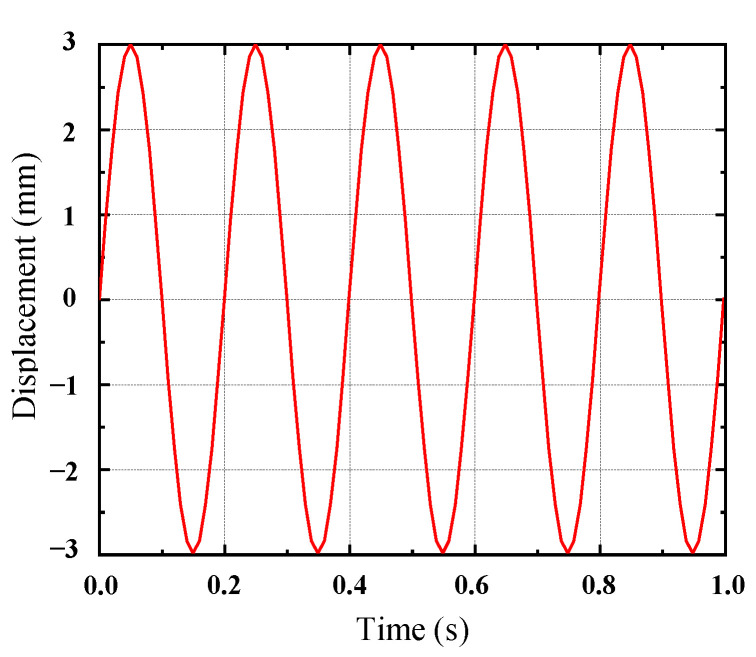
Harmonic excitation displacement time curve.

**Figure 6:**
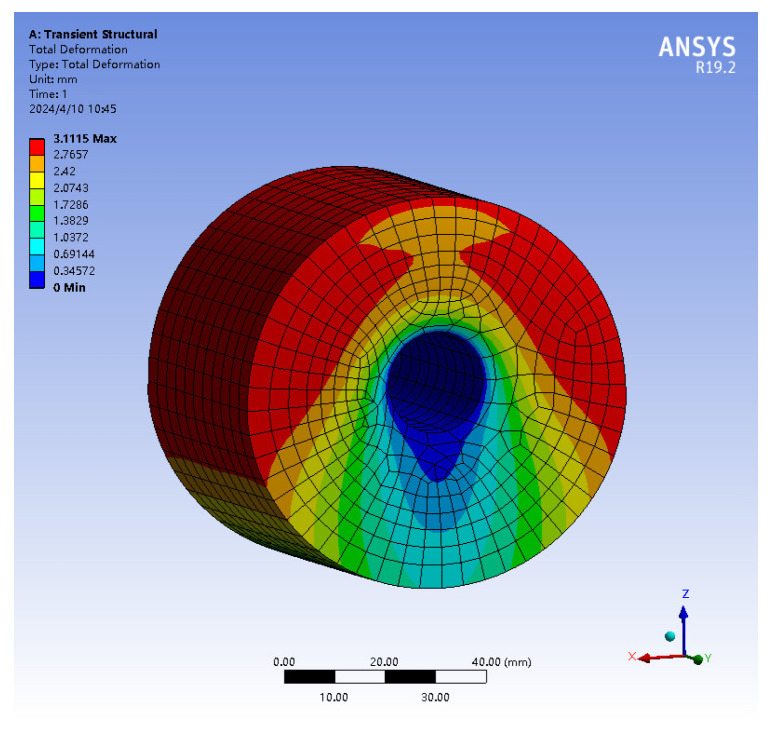
Deformation cloud map of rubber shock absorber compressed by 3 mm.

**Figure 7:**
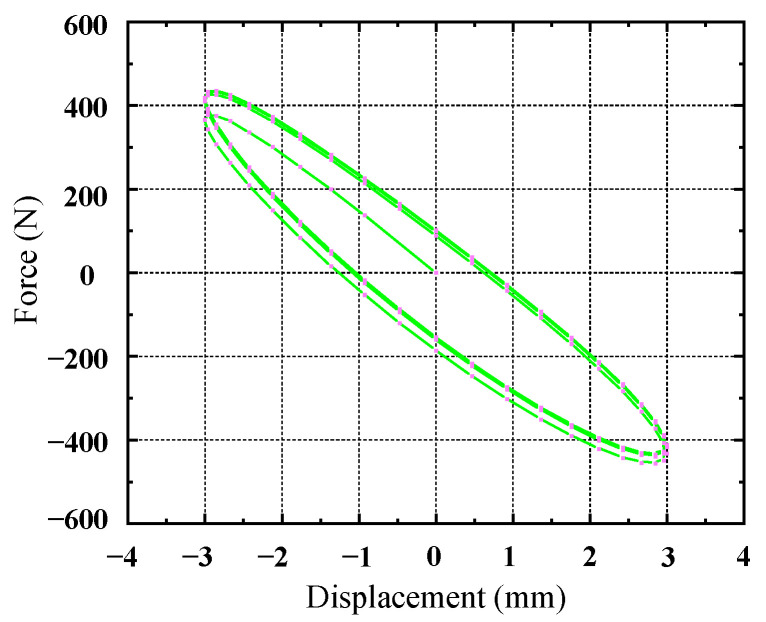
Hysteresis loop of rubber shock absorber for five cycles.

The 3D model of the rubber shock absorber was imported into ANSYS19.2, and it was meshed using SOLID186 elements. The rubber material properties were defined by selecting the Ogden third-order and Prony shear models from the material library, utilizing the constitutive parameters derived from Table 4 and Table 5. A harmonic excitation of 5 Hz was applied to the rubber damper using the transient analysis module. The center through-hole was fixed, and a force was applied to the outer surface of the rubber damper according to its actual installation specifications. The displacement loading curve, as indicated in Figure 5, was implemented. In the output settings, a deformation contour map of the rubber damper was generated, as presented in Figure 6. The maximum displacement of the loading surface was recorded, along with the dynamic reaction force at the fixed end, to create a hysteresis loop, which is illustrated in Figure 7.

As shown in Figure 7, due to the viscoelasticity of rubber, its displacement response lags behind the force response, forming an elliptical hysteresis loop that stabilizes during the second loading and unloading cycle. The hysteresis loop data obtained from the fifth cycle were recorded, and the damper’s stiffness, damping, and other parameters were calculated, as shown in Table 6.

**Figure 8:**
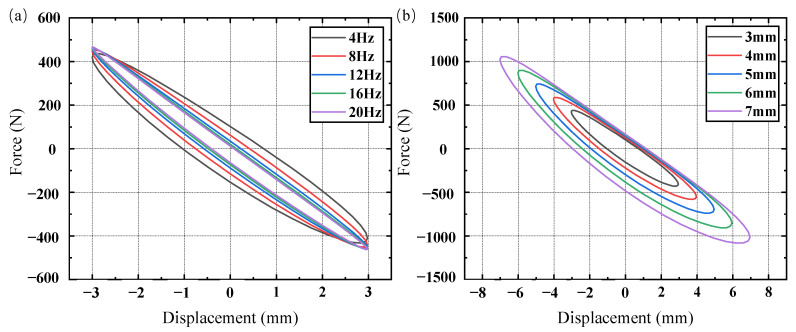
Rubber damper hysteresis loop: (**a**) different excitation frequencies; (**b**) different excitation amplitudes.

The stiffness and damping characteristics of rubber materials depend on the frequency and amplitude of the excitation load. To thoroughly examine these properties, harmonic excitations were applied to the rubber suspension with an amplitude of 3 mm at frequencies of 4 Hz, 8 Hz, 12 Hz, 16 Hz, and 20 Hz. Additionally, excitations with amplitudes of 3 mm, 4 mm, 5 mm, 6 mm, and 7 mm were applied at a frequency of 5 Hz. The hysteresis loop data for five cycles were calculated, as shown in Figure 8, and the data from the last cycle were used to determine the dynamic stiffness and damping of the rubber material, as shown in Table 7.

Table 7 shows that the dynamic stiffness and damping of rubber dampers increase with the excitation frequency and amplitude. Specifically, when the excitation frequency rises from 4 Hz to 20 Hz, the dynamic stiffness increases by 8.1% (from 143.5 N/mm to 155.12 N/mm), and the damping increases by 12.7% (from 1.11 N·s/mm to 1.2512 N·s/mm). Similarly, as the excitation amplitude increases from 3 mm to 7 mm, the dynamic stiffness increases by 3.7% (from 145.22 N/mm to 150.61 N/mm), and the damping increases by 15% (from 1.1522 N·s/mm to 1.3239 N·s/mm). These changes are due to the greater friction and molecular interactions within the rubber, which result in dynamic hardening and increased conversion of vibration energy into thermal energy, thereby enhancing the stiffness and damping of the rubber dampers.

Based on the above analysis, it is evident that the stiffness and damping of the rubber dampers increase with the excitation frequency and amplitude. However, the range of variation is relatively limited, leading to poor adaptability of rubber dampers to complex road surfaces. To tackle the issue of limited flexibility in adjusting stiffness and damping of rubber shock absorbers, a rubber-based shape–memory alloy (SMA) composite shock absorber was proposed. The objective is to examine the influence of loading rate on the stiffness and damping characteristics of the composite shock absorber, with the goal of ensuring efficient damping and energy dissipation within their designated super-elastic ranges.

### 3.2. Structural Design of Rubber-Based SMA Composite Material Vibration Dampers

**Figure 9:**
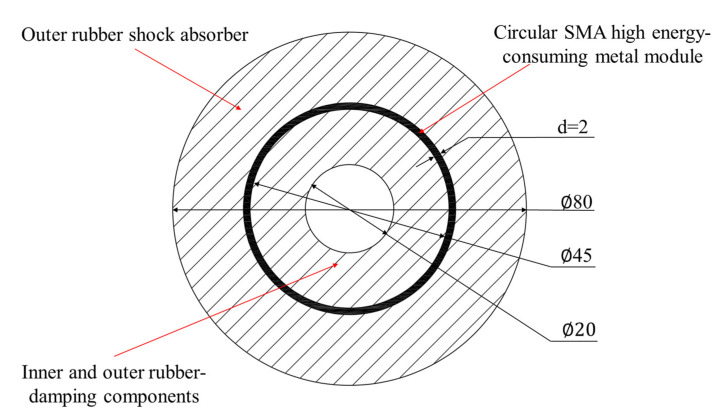
The cross section view of rubber-based SMA composite vibration damper.

In this section, we focus on commonly used sleeve-type shock absorbers, where the SMA, known for its increased stiffness and damping, is used as the support and energy dissipation material. Softer rubber is used for cushioning. This combination results in an SMA composite shock absorber with a rubber foundation. Through the parametric modeling and finite element simulation, we thoroughly examined the stiffness and damping properties of composite dampers under harmonic excitation with various loading rates and amplitudes. Figure 9 displays the structural profile of the SMA composite damper with a rubber foundation designed in this study.

For the convenience of subsequent simulation research, the DesignModeler module of ANSYS19.2 was used to parameterize the rubber-based SMA composite vibration damper. The structural parameters of the rubber-based SMA composite vibration damper are shown in Table 8, where d is the thickness of the circular SMA metal module, *H* is the overall width of the shock absorber, *D*1 is the inner diameter of the inner rubber-damping element, *D*2 and *D*3 are the inner and outer diameters of the SMA metal module, respectively, and *D*4 is the outer diameter of the outer-rubber-damping element.

To evaluate the stability of the structure, as well as the levels of vibration reduction and energy dissipation, the ANSYS19.2 transient analysis module was employed to simulate the composite damper. The material parameters of the inner and outer-rubber components are presented in Table 4 and Table 5, respectively. The material properties of the SMA metal module were integrated into a user-defined SMA-constitutive model subroutine using APDL instructions. When using the ANSYS19.2 UPFs secondary development tool to customize the material constitutive models, the usermat. f user subroutine is mainly used. By calling the usermat3d.f subroutine, one reads the state variables of a certain integration point in the current iteration step, including stress, strain, time increment, etc.; based on the constitutive model of the defined material, calculate the Jacobian matrix (D = ∂Δσ/∂Δε), and update the stress, strain, Jacobian matrix, and other data of the current integration point, by combining the calculated Jacobian matrix and the read state variables, and return the results to the ANSYS19.2 main program.

During the compilation process of the subroutine, the Jacobian matrix needs to be calculated through the increment of stress and strain, as shown in Equation (6). The increment formula is as follows:

(6) $$\Delta \sigma_{ij}=\left\{\begin{array}{c} \frac{\Delta \varepsilon_{ij}}{\Delta t}\cdot \left(-2.49\times 10^{6}\cdot \dot{\varepsilon }\cdot \varepsilon^{2}+2.57\times 10^{5}\cdot \dot{\varepsilon }\cdot \varepsilon -1.34\times 10^{3}\cdot \dot{\varepsilon }\right)\;Loading\\ \frac{\Delta \varepsilon_{ij}}{\Delta t}\cdot \left(-1.69\times 10^{6}\cdot \dot{\varepsilon }\cdot \varepsilon^{2}+7.38\times 10^{5}\cdot \dot{\varepsilon }\cdot \varepsilon -52.2\times 10^{3}\cdot \dot{\varepsilon }\right)\;Unloading \end{array}\right.$$

The main diagonal elements of its Jacobian matrix are i = 1~6:

(7) $$D_{ii}=\left\{\begin{array}{c} \frac{-2.49\times 10^{6}\cdot {\varepsilon_{i}}^{2}+2.57\times 10^{5}\cdot \varepsilon_{i}-1.34\times 10^{3}}{\Delta t}\;Loading\\ \frac{-1.69\times 10^{6}\cdot {\varepsilon_{i}}^{2}+7.38\times 10^{5}\cdot \varepsilon_{i}-52.2\times 10^{3}}{\Delta t}\;Unloading \end{array}\right.$$

**Figure 10:**
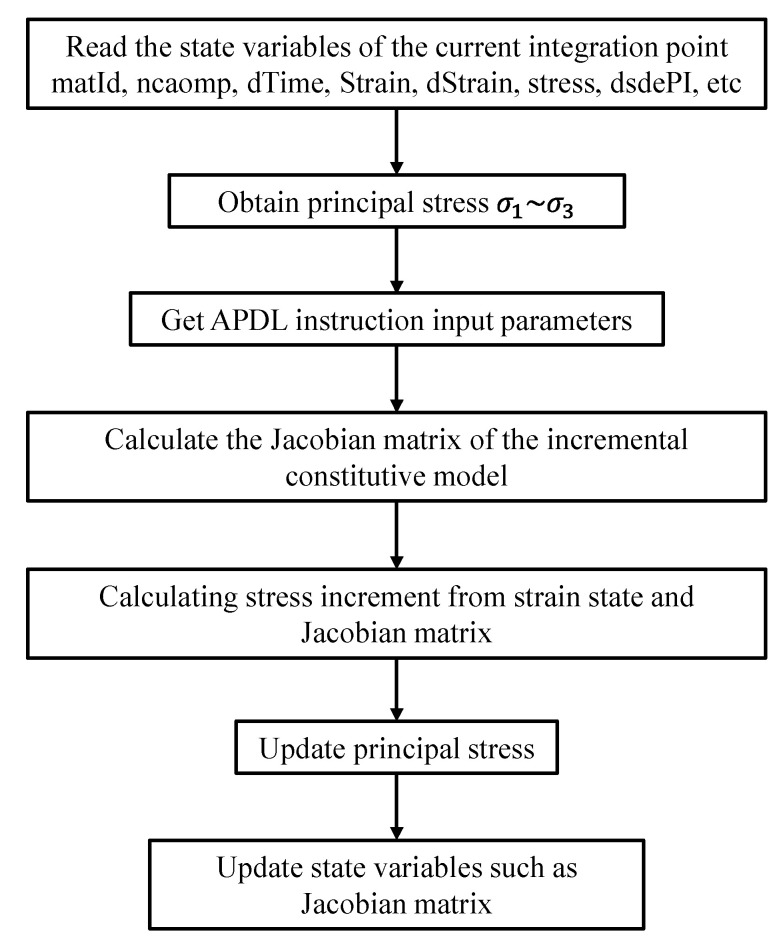
User-defined subroutine structure framework for SMA.

The structure framework of the custom subroutine is shown in Figure 10.

After completing the user-defined program definition, the user-defined functions were imported using the Workbench Mechanical module’s User Programmable Features module, and instructions were added using the SMA geometry module. APDL instructions are to be created so that they invoke user-defined functions and perform computations during the program’s execution, based on the user-defined SMA-constitutive model.

**Figure 11:**
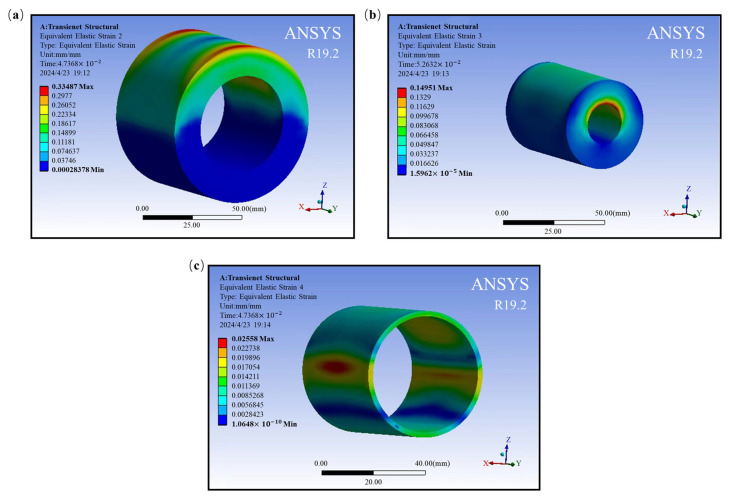
Strain cloud map: (**a**) outer-rubber shock absorber; (**b**) inner-rubber-damping components; (**c**) SMA metal module.

Fixed support was applied to the central through-hole, and frictional contact was established on the interface between the inner and outer-rubber-damping components and the elastic SMA module, with a friction coefficient of 0.15. The required parameters for defining the SMA material properties through APDL are determined based on the literature [31], as shown in Table 9. Figure 11 shows the strain cloud diagram of the SMA metal module and rubber-damping component at the end of the loading stage.

Here, μ is the Poisson’s ratio; SAS, FAS, SSA, and FSA represent the starting and ending stresses of martensitic and austenitic transformations, respectively; eL is the maximum strain; alpha is the coefficient of determination for tension and compression; EA is the elastic modulus of austenite; and ES is the elastic modulus of martensite.

From Figure 11, it can be seen that the maximum strain of the rubber-damping component is 0.33487, and the maximum strain of the circular SMA metal module is 0.02558. During the compression stage, the outer rubber first undergoes compression deformation and transmits the external load to the SMA metal module through compressive stress. Due to the large elastic modulus of the SMA material, the stiffness of the SMA metal module is higher than that of the outer-rubber-damping ring, and the deformation is relatively small, resulting in gaps between the SMA layer and the outer rubber. Through deformation, the SMA metal module further transfers external loads to the inner rubber; nevertheless, the inner rubber’s deformation space is constrained. The majority of the stress is used to compress and distort the rubber material in the y direction, while the residual stress deforms the inner rubber in the z direction. Overall, in this model, stress and strain are transmitted through the interaction between the rubber and SMA metal modules, and the characteristics of each component collectively affect the overall performance of the composite shock absorber.

**Figure 12:**
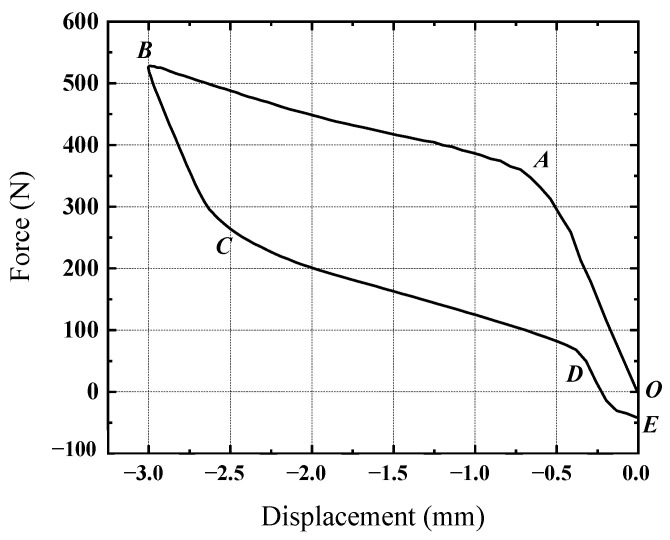
Force displacement curve of rubber-based SMA composite shock absorber during loading and unloading process.

Due to the symmetrical structure of the sleeve-type shock absorber, the force displacement curves of the upper-surface compression stage during the loading and unloading processes are drawn as shown in Figure 12.

As shown in Figure 12, the force-displacement curve of the composite damper exhibits characteristics of both the viscoelastic damping energy dissipation of the rubber material and the phase change energy dissipation of the SMA material. The hysteresis loop can be divided into segments OA, AB, BC, CD, and DE during the compression loading stroke. The OA segment primarily consists of the super-elastic region of the rubber and the linear elastic region of the SMA. The AB segment is mainly due to the (Austenite, A)→(Martensite, M) phase transition of the SMA metal module, which reduces the material’s elastic modulus and overall stiffness, thereby slowing the upward trend of the curve. During the compression-unloading stroke, the BC segment involves martensitic linear elastic unloading and the hysteresis loop unloading process, formed by the viscoelasticity of the rubber materials. As the displacement decreases, the elastic modulus of the material increases in the CD segment due to the (Martensite, M)→(Austenite, A) phase transition of the SMA ring. In the DE segment, due to the viscoelastic effect of the rubber, although the deformation returns to zero, residual stress remains in the rubber-damping component, generating a reverse rebound force due to the action of multiple molecules. This rebound force must be gradually dissipated through multiple compression and recovery processes to reach the initial state of zero stress.

Due to the composite shock absorber being composed of two materials, SMA and rubber, the hysteresis loop will change at different strain rates and frequencies. For the convenience of subsequent research, the ratio of the maximum dynamic reaction force to the maximum displacement of the hysteresis loop is used to characterize the dynamic stiffness. The dissipation energy and damping values of the composite shock absorber are calculated as shown in Table 10.

It is evident from Table 10 that the stiffness and damping of the damper increase when a circular SMA metal module is embedded in rubber materials. Nevertheless, the high stiffness and damping are not the primary goals of damper design. Instead, the stiffness and damping should be optimized to suit various working conditions, such as different excitation rates or amplitudes. To further investigate the energy dissipation characteristics of the rubber-based SMA composite dampers, the influence of harmonic excitation loading rate and excitation amplitude on the stiffness and damping of composite dampers were explored based on the established parameterized model.

### 3.3. Stiffness and Damping Characteristics of Composite Dampers under Different Excitation Conditions

**Figure 13:**
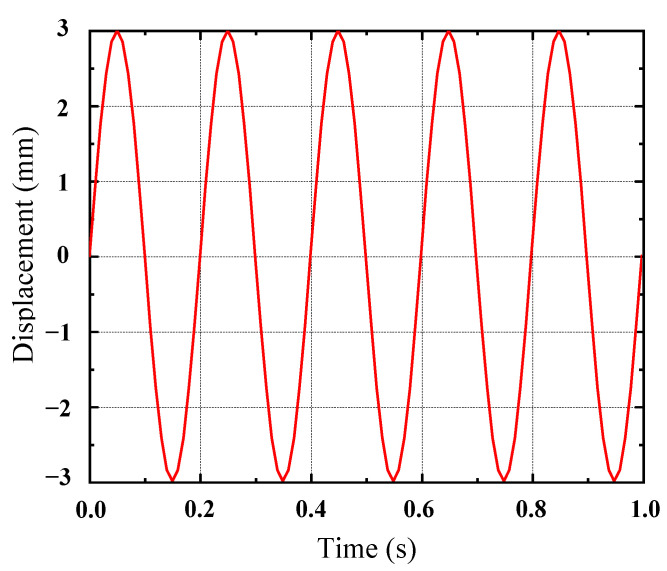
Harmonic excitation at different loading rates.

**Figure 14:**
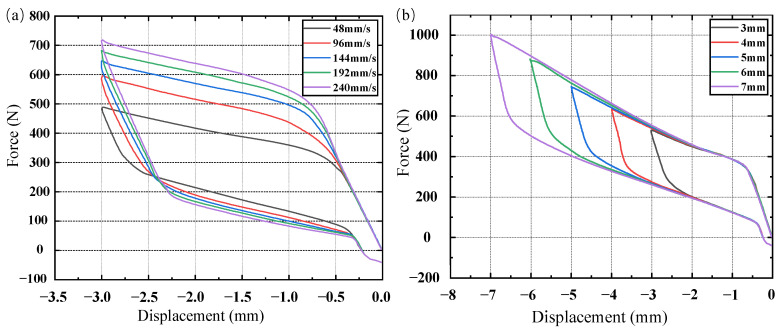
Force–displacement curve of rubber-based SMA composite vibration damper during loading and unloading process: (**a**) different loading rates; (**b**) different excitation amplitudes.

Variations in the road conditions induce diverse excitations on the cab, affecting the strain rate and amplitude of the shock absorber. Typically, the strain amplitude correlates with displacement excitation amplitude, while the strain rate is influenced by the shock absorber’s loading rate. To explore the impact of the loading rate and excitation amplitude on the phase transition and stiffness–damping characteristics of SMA, rubber-based SMA composite dampers underwent harmonic excitations at loading rates of 48 mm/s, 96 mm/s, 144 mm/s, 192 mm/s, and 240 mm/s, all with an excitation amplitude of 3 mm. Additionally, excitations with amplitudes of 3 mm, 4 mm, 5 mm, 6 mm, and 7 mm and a loading rate of 75 mm/s were applied. The loading rate was computed by dividing the amplitude of the harmonic excitation by one-quarter of its period. For example, Figure 13 illustrates the displacement–time curve for harmonic excitation with a loading rate of 48 mm/s, while Figure 14 presents the force–displacement curves under various loading rates and excitation amplitudes.

Figure 14 shows that as the strain rate increases, the area enclosed by the force–displacement curve significantly increases during the deformation of the SMA metal module. This indicates that the rubber-based SMA composite vibration dampers have a higher dissipation capacity at high loading rates. Small loading displacements result in minor SMA strains that do not trigger martensitic transformation, and the loading rates have little effect on the stiffness of the rubber material or the austenite structure. Therefore, the force–displacement curves of the composite damper at different loading rates are nearly identical. At large displacements, the rubber-damping component shows continuous stress changes due to its viscoelasticity, while the SMA metal module undergoes significant stress changes due to austenite and martensite transformations. Consequently, the force–displacement curve of the overall structure reflects the combined characteristics of both SMA and rubber materials. During the unloading stage with small displacements, the SMA is in the linear unloading process of austenite, and the rubber dampers’ displacement response lags behind the force response due to viscoelasticity. Thus, the force–displacement curve of the composite dampers mainly shows the hysteresis characteristics of the rubber materials. As the excitation amplitude increases, the area enclosed by the force–displacement curve significantly increases, indicating that the rubber-based SMA composite dampers have greater dissipation capacity under large load excitations.

**Figure 15:**
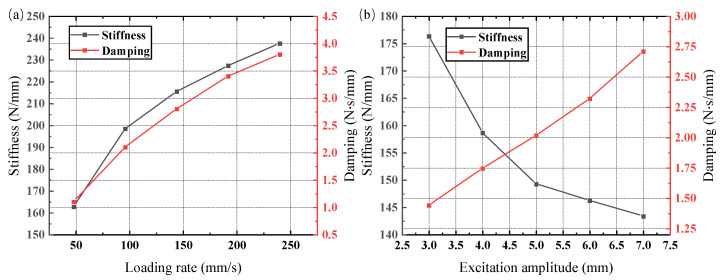
Curve of stiffness and damping characteristics of rubber-based SMA composite shock absorber with loading rate and excitation amplitude: (**a**) loading rate; (**b**) excitation amplitude.

The stiffness, damping, and dissipation energy were calculated for each loading rate and excitation amplitude. The results are presented in Table 11, and Figure 15 shows the variation curves of stiffness and damping with the loading rate and excitation amplitude.

Figure 15 illustrates how the stiffness and damping of the shock absorber increase in tandem with the harmonic loading rate. As the excitation amplitude increases, the shock absorber’s stiffness gradually decreases, while its damping gradually increases. This is because the force–displacement curve characteristics of the overall structure tend to resemble those of SMA materials, due to the relatively low stiffness of rubber-damping components.

When the excitation amplitude is small, the SMA metal module remains in an austenitic state with a high elastic modulus. As the amplitude increases, the SMA module undergoes martensitic transformation, leading to a mixed structure of austenite and martensite phases, and a decrease in elastic modulus. Consequently, within the super-elastic range of SMA, the larger the excitation amplitude, the closer the overall stiffness of the structure approaches that of the mixed phases. Thus, the overall stiffness of the composite damper decreases with increased excitation amplitude. On the other hand, the damping of both SMA and rubber materials increases with higher excitation amplitudes, thereby causing the overall damping of the structure to also increase.

## 4. Conclusions and Prospects

Listed below are this article’s main conclusions:

1. Based on the stress–strain curves obtained for shape–memory alloys (SMAs) at varying strain rates, a stress increment expression was determined through rigorous regression analysis and fitting, considering the influence of the loading rate. This effort resulted in updating the stress calculation formula, specifically for the super-elastic phase transition process of SMAs and improving the Auricchio constitutive model. This provides researchers with a more accurate tool to describe the behavior of SMA materials, which helps to gain a deeper understanding of the material properties of SMA.
2. A user-defined subroutine for the enhanced SMA-constitutive model was developed, and the impact of the harmonic excitation loading rate on the stiffness and damping properties of rubber dampers, using the finite element simulation, was investigated. This study offers empirical evidence for guiding the structural design of rubber-based SMA composite dampers.
3. A rubber-based SMA composite vibration damper was proposed, leveraging SMA for enhanced stiffness and damping to support the energy dissipation, alongside softer rubber acting as a buffer. The stiffness and damping characteristics of the composite vibration damper were comprehensively analyzed under the harmonic excitation with varying loading rates and amplitudes, employing parametric modeling and finite element simulation. This provides practitioners with an idea for designing composite shock absorbers with adjustable stiffness and damping.

The limitations and prospects of this article are as follows:

1. This study primarily concentrates on specific types of sleeve-type shock absorbers, and the applicability of the constitutive model might require further validation for other types of shock absorber structures. Further extending the applicability of the constitutive model and investigating its application in diverse types of shock absorbers and structures will constitute the focal point of the subsequent research.
2. This article primarily takes into account the impacts of the loading rate, excitation amplitude, and excitation frequency on the stiffness and damping characteristics of the shock absorber. However, in practical applications, more intricate working conditions may be encountered, thereby necessitating a further expansion of the research scope to more accurately depict the performance of the composite shock absorbers under the actual operating conditions.
3. This article merely offers a preliminary exploration of the interaction mechanism between the SMA and rubber materials, hence further theoretical analysis and experimental research are required to unveil their inherent laws.

## Figures and Tables

**Table 1:** Fitting coefficients corresponding to different strain rates at different stages.

Condition	Strain Rate (/s)	Quadratic Coefficient a	Coefficient of First-Order Term b	Intercept c	Goodness of Fit R^2^
Loading	0.0042	−4475.95	599.7476	1.396	0.98819
	0.0083	−22,556.2	2465.067	−9.66657	0.97522
	0.0125	−31,775.7	3291.25	−22.771	0.9351
	0.0167	−43,860	4631.25	−27.4979	0.93463
	0.0210	−56,659.9	5690.137	−23.8451	0.93117
	0.0250	−57,018	5912.142	−32.1167	0.94121
Unloading	−0.0042	12,603.69	−887.377	0.23807	0.89597
	−0.0083	15,977.38	−706.671	−25.2016	0.91442
	−0.0125	25,733.81	−988.619	−57.1296	0.95415
	−0.0167	28,284.76	−1090.21	−46.3745	0.96554
	−0.0210	36,877.74	−1919.07	−29.4848	0.95075
	−0.0250	35,535.54	−1619.25	−40.1179	0.94014

**Table 2:** Linear fitting coefficients of different fitting parameters and strain rates at different stages.

Condition	Quadratic Coefficient a	Coefficient of First-Order Term b	Intercept c	Intercept d
Loading	−2.49 × 10^6^	2.57 × 10^5^	−1.34 × 10^3^	0
Unloading	−1.69 × 10^6^	7.38 × 10^5^	−52.2	0

**Table 3:** Structural parameters of rubber shock absorbers.

Through-Hole Diameter $D_{1}$ (mm)	Outer Diameter $D_{2}$ (mm)	Width H (mm)
20	80	55

**Table 4:** Odgen third-order hyper-elastic constitutive parameters [30].

$\mu_{1}$	$\alpha_{1}$	$\mu_{2}$	$\alpha_{2}$	$\mu_{3}$	$\alpha_{3}$	$D_{1}$
6.1803	1.3	0.0118	5	−0.0981	−2	0.01

**Table 5:** Prony class 2 viscoelastic constitutive parameters [30].

$G_{1}$	$\tau_{1}$	$G_{2}$	$\tau_{2}$
0.4	0.05	0.6	0.1177

**Table 6:** Parameters related to the hysteresis loop of the fifth cycle of rubber shock absorbers.

Maximum Displacement (mm)	Maximum Dynamic Reaction Force (N)	Dynamic Stiffness (N/mm)	Damping (N∙s/mm)	Hysteresis Dissipation Energy (N∙mm)
3	435.67	145.2233	1.1522	605.0739

**Table 7:** Dynamic stiffness, damping, and hysteresis dissipation energy of rubber dampers under different excitation frequencies and amplitudes.

Harmonic Excitation	Numerical Value	Dynamic Stiffness (N/mm)	Damping (N∙s/mm)	Hysteresis Dissipation Energy (N∙mm)
Frequency (Hz)	4	143.4563	1.11	635.0062
	8	151.2161	1.1765	419.1714
	12	153.6465	1.2026	291.2865
	16	154.4906	1.2298	221.9129
	20	155.1190	1.2512	178.9395
Amplitude (mm)	3	145.2233	1.1522	605.0739
	4	146.5077	1.193336	1093.4
	5	147.8468	1.228092	1737.9
	6	149.2220	1.266672	2551.8
	7	150.6103	1.3239	3549.4

**Table 8:** Structural parameters of circular ring rubber-based SMA composite vibration dampers.

D1	D2	D3	D4	d	H
20	45	49	80	2	55

**Table 9:** SMA material parameters [31].

EA (Mpa)	ES (Mpa)	μ	SAS (Mpa)	FAS (Mpa)	SSA (Mpa)	FSA (Mpa)	eL	alpha
30,000	30,000	0.36	520	600	300	200	0.07	0

**Table 10:** Stiffness, damping, and dissipation energy of rubber-based SMA composite shock absorbers.

Stiffness (N/mm)	Damping (N·s/mm)	Dissipated Energy (N·mm)
176.33	1.4408	639.8969

**Table 11:** Stiffness, damping, and dissipation energy of rubber-based SMA composite shock absorbers under different loading rates and excitation amplitudes.

Incentive Conditions	Numerical Value	Dynamic Stiffness (N/mm)	Damping (N∙s/mm)	Hysteresis Dissipation Energy (N∙mm)
Loading rate (mm/s)	4	162.7308	1.1	562.1129
	8	198.4387	2.1	639.8969
	12	215.5458	2.8	795.9158
	16	227.3613	3.4	930.6663
	20	238.5336	3.8	1010.6
Excitation amplitude (mm)	3	176.33	1.4408	639.8969
	4	158.6251	1.7456	923.0231
	5	149.2551	2.0171	1229.3
	6	146.2201	2.3198	1618.6
	7	143.3479	2.7093	2090.2

## Data Availability

The original contributions presented in the study are included in the article, further inquiries can be directed to the corresponding author.
